# Clinical Trial: *CYP2D6* Related Dose Escalation of Tamoxifen in Breast Cancer Patients With Iranian Ethnic Background Resulted in Increased Concentrations of Tamoxifen and Its Metabolites

**DOI:** 10.3389/fphar.2019.00530

**Published:** 2019-05-24

**Authors:** Zahra Khalaj, Zohreh Baratieh, Parvaneh Nikpour, Matthias Schwab, Elke Schaeffeler, Fariborz Mokarian, Hossein Khanahmad, Rasoul Salehi, Thomas E. Mürdter, Mansoor Salehi

**Affiliations:** ^1^Department of Genetics and Molecular Biology, Faculty of Medicine, Isfahan University of Medical Sciences, Isfahan, Iran; ^2^Child Growth and Development Research Center, Research Institute for Primordial Prevention of Non-communicable Disease, Isfahan University of Medical Sciences, Isfahan, Iran; ^3^Dr. Margarete Fischer-Bosch-Institute of Clinical Pharmacology, Stuttgart, Germany; ^4^Department of Clinical Pharmacology, University Hospital Tübingen, Tübingen, Germany; ^5^Department of Pharmacy and Biochemistry, University Hospital Tübingen, Tübingen, Germany; ^6^University of Tübingen, Tübingen, Germany; ^7^Cancer Prevention Research Center, Isfahan University of Medical Sciences, Isfahan, Iran; ^8^Cellular, Molecular and Genetics Research Center, Isfahan University of Medical Sciences, Isfahan, Iran

**Keywords:** *CYP2D6*, dose, endoxifen, genotype, metabolite, tamoxifen

## Abstract

**Introduction:** The polymorphic enzyme cytochrome P450 2D6 (CYP2D6) catalyzes a major step in the bioactivation of tamoxifen. Genotyping of clinically relevant *CYP2D6* alleles and subsequent dose adjustment is a promising approach to individualize breast cancer therapy. The aim of this study was to investigate the relationship between the plasma levels of tamoxifen and its metabolites and different *CYP2D6* genotypes under standard (20 mg/day) and dose-adjusted therapy (Registration ID in Iranian Registry of Clinical Trials: IRCT2015082323734N1).

**Materials and Methods:** Using TaqMan^®^ assays common alleles of *CYP2D6* (^∗^1, ^∗^2, ^∗^4, ^∗^5, ^∗^6, ^∗^10, ^∗^17, and ^∗^41) and gene duplication were identified in 134 breast cancer patients. Based on *CYP2D6* genotypes patients with an activity score 1 (*n* = 15) and 0–0.5 (*n* = 2) were treated with tamoxifen adjusted dosage of 30 and 40 mg/day, respectively. The concentration of tamoxifen and its metabolites before and after 4 and 8 months of dose adjustment were measured using LC-MS/MS technology.

**Results:** At baseline, (Z)-endoxifen plasma concentrations (33 ± 15.5, 28.1 ± 14, 26.6 ± 23.4, 14.3 ± 8.6, and 10.7 ± 5.5 nmol/l for EM/EM, EM/IM, EM/PM, IM/IM and PM/PM, respectively) and the metabolic ratio (Z)-Endoxifen/N-desmethyltamoxifen (0.0558 ± 0.02, 0.0396 ± 0.0111, 0.0332 ± 0.0222, 0.0149 ± 0.0026, and 0.0169 ± 0.0177 for EM/EM, EM/IM, EM/PM, IM/IM, and PM/PM, respectively) correlated with *CYP2D6* genotype (Kruskal–Wallis *p* = 0.013 and *p* < 0.0001, respectively). Dose escalation to 30 and 40 mg/day in patients with a CYP2D6 activity score of 1 (*n* = 15) and 0–0.5 (*n* = 2) resulted in a significant increase in (Z)-endoxifen plasma levels (22.17 ± 24.42, 34.43 ± 26.54, and 35.77 ± 28.89 nmol/l at baseline, after 4 and 8 months, respectively, Friedman *p* = 0.0388) along with the plasma concentrations of tamoxifen and its other metabolites. No severe side effects were recorded during dose escalation.

**Conclusion:** For the first time, we show the feasibility of dose escalation of tamoxifen in breast cancer patients with compromised CYP2D6 activity and Iranian ethnic background to increase the plasma concentrations of (Z)-endoxifen.

## Introduction

Breast cancer is one of the most common types of cancer and the leading cause of cancer related death in women worldwide (Torre et al., 2015). More than 70% of affected patients are ER-positive and therefore eligible for adjuvant endocrine therapy with tamoxifen (Early Breast Cancer Trialists’ Collaborative Group, 2005; Howlader et al., 2014). This drug has been the gold standard treatment of ER-positive breast cancer patients for more than three decades (Jordan, 2014). Tamoxifen acts as an estrogen antagonist and thus inhibits ER-dependent cell proliferation in breast cancer cells (Jordan, 1976). Tamoxifen decreases the risk of recurrence and mortality by 50 and 30%, respectively. Even so, the efficacy of tamoxifen shows a high degree of variation, with 30–50% of patients exhibiting drug resistance (Early Breast Cancer Trialists’ Collaborative Group, 2005).

Tamoxifen is extensively metabolized by several members of the cytochrome P450 enzymes (Desta et al., 2004; Brauch et al., 2009). Some of the resulting metabolites have higher anti-estrogenic effects in breast cancer tissue than tamoxifen itself. Among these, 4-hydroxytamoxifen (4-OH-Tam) and 4-hydroxy-N-desmethyl-tamoxifen (endoxifen) show the highest anti-estrogenic potency (Lim et al., 2006). As the steady state plasma concentration of endoxifen is approx. 10-times higher compared with levels of 4-OH-Tam, endoxifen is considered to be the major active tamoxifen metabolite (Stearns et al., 2003).

**FIGURE 1:**
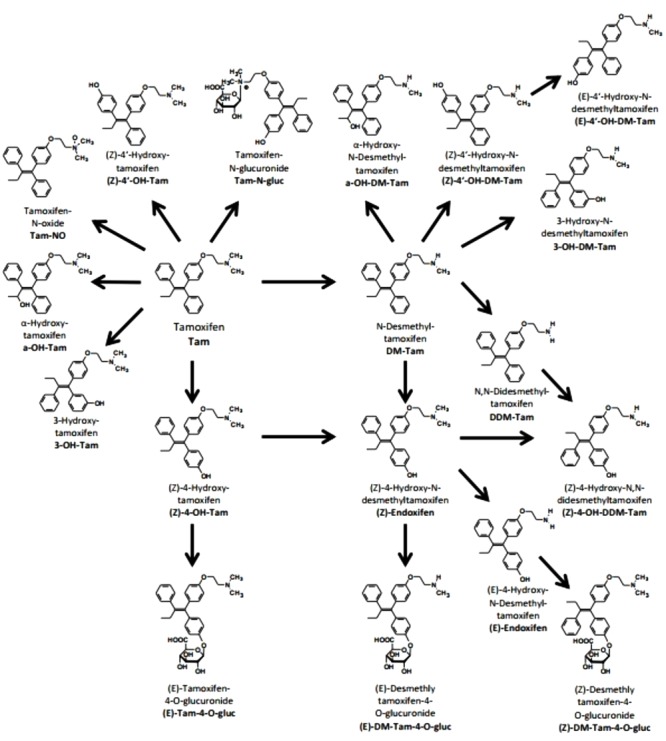
Metabolism of tamoxifen. Structures, names, and abbreviations of all metabolites which were quantified in the present study are shown (Brauch et al., 2009).

The bioactivation of tamoxifen to its main active metabolite endoxifen comprises two permutable steps, namely N-desmethylation and 4-hydroxylation (Figure 1; Brauch et al., 2009). The rate limiting step in the major route is the 4-hydroxylation via CYP2D6 (Desta et al., 2004).

CYP2D6 is encoded by a highly polymorphic gene with more than 100 different alleles (Sim and Ingelman-Sundberg, 2010). It has been shown that a number of its genetic variants affect the activity of the enzyme and may decrease the levels of the active metabolites and thus result in decreased therapeutic response to tamoxifen (Goetz et al., 2005; Nowell et al., 2005; Schroth et al., 2009). According to the enzyme activity, four different phenotypic alleles including PM, IM, EM, and UM have been categorized (Bradford, 2002; Daly, 2003; Zanger et al., 2004; van Schaik, 2005; McGraw and Waller, 2012). There is a body of evidence that subjects carrying dysfunctional alleles of *CYP2D6* have less benefit from tamoxifen therapy because of lower plasma concentrations of the active metabolite endoxifen (Jin et al., 2005; Lim et al., 2007; Madlensky et al., 2011; Karle et al., 2013; Saladores et al., 2015).

The frequency of *CYP2D6* alleles varies in different populations. Various studies indicate that the prevalence of normal alleles (^∗^1 and ^∗^2) in the European population is around 70%. Twenty-six percent of the population in Europe is carrying one PM (^∗^3, ^∗^4, ^∗^5, and ^∗^6) allele and the most common PM allele is *CYP2D6*^∗^4. However, the frequency of PM alleles in the Asian population is less than 1%. In contrast, in the Asian population between the reduced function alleles (^∗^10, ^∗^17, and ^∗^41), *CYP2D6*^∗^10 is significantly more frequent by about 40–50% compared to Europeans (Bradford, 2002).

Regarding the Iranian population and the ethnic background few studies indicate the most relevant *CYP2D6* alleles (Kouhi et al., 2009; Hashemi-Soteh et al., 2011; Bagheri et al., 2015). Accordingly, we decided to genotype the breast cancer patients of our study for the *CYP2D6* alleles *^∗^1, ^∗^2, ^∗^4, ^∗^5, ^∗^6, ^∗^10, ^∗^17*, and *^∗^41* as well as the gene duplication to comprehensively cover *CYP2D6* alleles representative of the Iran population (Isfahan province). Based on their *CYP2D6* alleles, the patients were assigned to different genotype groups discriminating for CYP2D6 phenotypes by CYP2D6 activity scores (AS) (Gaedigk et al., 2008). Plasma concentrations of tamoxifen and its metabolites following a standard tamoxifen dose of 20 mg/day were correlated with CYP2D6 phenotype. In patients with an activity score of one or less, the standard tamoxifen dose was adjusted accordingly to 40 mg. Follow-up after 4 and 8 months after dose adjustment was performed and plasma levels of tamoxifen and its metabolites as well as adverse events were assessed.

## Materials and Methods

### Patients

A total of 134 patients diagnosed with estrogen and/or progesterone receptor positive breast cancer, were enrolled in the Breast Cancer Research Center in Isfahan province, Iran. The main characteristics of the patients incorporated in the study, are presented in Table 1. Peripheral blood (5 ml) was collected from each patient in sterile tubes containing EDTA. A fixed time of blood collection was waived, since PBPK simulations showed only small fluctuations of endoxifen plasma levels within the dosing interval (Klopp-Schulze et al., 2018). The study was approved by the Ethical Committee of Isfahan University of Medical Sciences and written informed consent was obtained from all patients.

**Table 1:** The main characteristics of the patients.

Characteristics	All patients enrolled Number of patients (%) *N* = 134 (100)	Patients receiving 20 mg/day Number of patients (%) *N* = 117 (100)	Patients receiving 30 or 40 mg/day Number of patients (%) *N* = 17 (100)
**Age (years)**			
Median	45	45	46
Range	28–71	28–71	34–68
**BMI**			
Median	22	22	23
Range	18–32	18–32	19.5–30
**Menopausal status**			
Premenopausal	60 (44.8)	57 (48.7)	3 (17.7)
Postmenopausal	70 (52.2)	56 (47.9)	14 (82.3)
Unknown	4 (3.0)	4 (3.4)	0 (0.0)
**Chemotherapy**			
Yes	116 (86.6)	100 (85.5)	16 (94.1)
No	15 (11.2)	14 (12.0)	1 (5.9)
Unknown	3 (2.2)	3 (2.5)	0 (0.0)
**Radiation**			
Yes	122 (91.1)	105 (89.7)	17 (100)
No	9 (6.7)	9 (7.7)	0 (0.0)
Unknown	3 (2.2)	3 (2.6)	0 (0.0)
**Duration of tamoxifen use**			
>4 months, ≤2 years	81 (60.4)	71 (60.7)	10 (58.8)
>2 years	53 (39.6)	46 (39.3)	7 (41.2)
**Tumor size (cm)**			
≤2	33 (24.6)	31 (26.5)	2 (11.8)
2.1–5	67 (50.0)	57 (48.7)	10 (58.8)
>5	11 (8.2)	9 (7.7)	2 (11.8)
Unknown	23 (17.2)	20 (17.1)	3 (17.6)
**Grading**			
G1	16 (11.9)	15 (12.8)	1 (5.9)
G2	62 (46.3)	54 (46.2)	8 (47.1)
G3	32 (23.9)	31 (26.5)	1 (5.9)
Unknown	24 (17.9)	17 (14.5)	7 (41.2)
**Nodal status**			
Negative	51 (38.0)	42 (35.9)	9 (52.9)
Positive	62 (46.3)	56 (47.9)	6 (35.3)
Unknown	21 (15.7)	19 (16.2)	2 (11.8)
**Estrogen receptor status**			
Positive	127 (94.8)	111 (94.9)	16 (94.1)
Negative	2 (1.5)	2 (1.7)	0 (0.0)
Unknown	5 (3.7)	4 (3.4)	1 (5.9)
**Progesterone receptor status**			
Positive	121 (90.3)	105 (89.8)	16 (94.1)
Negative	6 (4.5)	6 (5.1)	0 (0.0)
Unknown	7 (5.2)	6 (5.1)	1 (5.9)
**HER-2 status**			
Positive	16 (11.9)	14 (12.0)	2 (11.8)
Negative	106 (79.1)	94 (80.3)	12 (70.6)
Unknown	12 (9.0)	9 (7.7)	3 (17.6)

*BMI, Body mass index; ER, estrogen receptor; PR, progesterone receptor; HER2, human epidermal growth factor receptor 2.*

### Genotyping

Genomic DNA was extracted from 200 μl of a peripheral blood sample of each patient using QIAamp DNA blood mini kit (Qiagen, Hilden, Germany). All patients included in this study were genotyped for *CYP2D6*^∗^2, ^∗^4, ^∗^6, ^∗^10, ^∗^17, and ^∗^41 alleles by TaqMan^®^ allelic discrimination assays (Applied Biosystems, Foster City, CA, United States) according to the manufacturer’s instructions. Determination of *CYP2D6* gene deletion (*CYP2D6^∗^5*) and duplication was performed using a pre-developed TaqMan^®^ *CYP2D6* Copy Number Assay (Hs00010001_cn, Thermo Fisher Scientific) and as a reference the TaqMan^®^ Copy Number Reference Assay RNase P (Thermo Fisher Scientific) as previously described (Schroth; Front Pharmacol 2017). Real-time PCR analyses were carried out according to the manufacturer’s instructions on a 7900 Real-Time PCR System (Thermo Fisher Scientific) and data were analyzed with the SDS2.4 software. In the present study, alleles are assigned according to the nomenclature for human *CYP2D6* alleles as previously published (Sim and Ingelman-Sundberg, 2010). Samples with one loss of function or reduced activity allele and a copy number of 3 were assigned EM phenotype. The allele frequencies were tested for deviation from the Hardy Weinberg equilibrium using the exact test^1^.

### Clinical Trial Design and Endpoints

**FIGURE 2:**
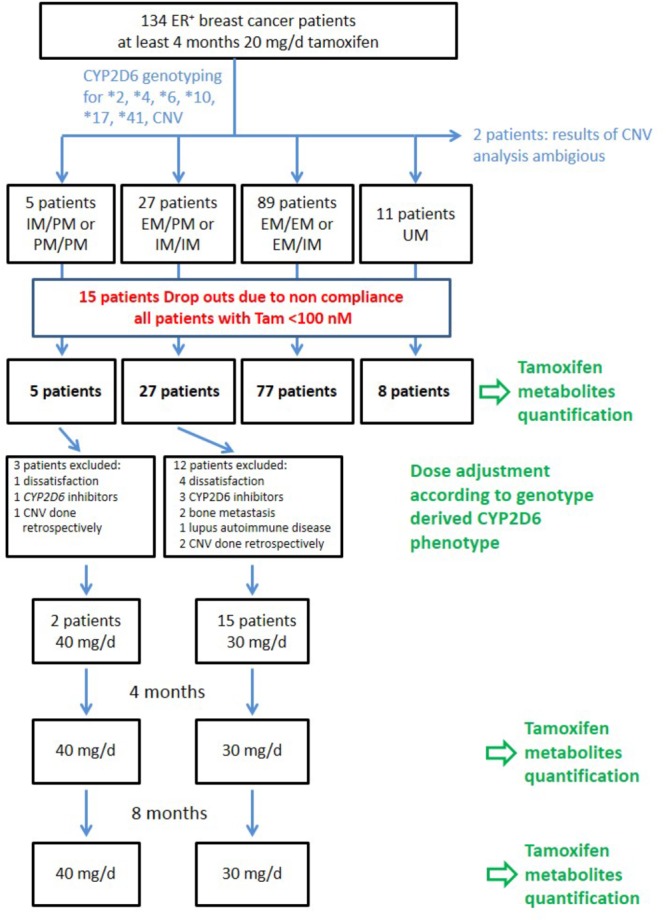
Flow chart of patients’ enrollment and sampling during the clinical trial.

Pre- and postmenopausal breast cancer patients (*n* = 134) who were on standard tamoxifen therapy of 20 mg daily for at least 4 months with normal liver and kidney function were enrolled. Exclusion criteria as defined by the SmPC were pregnancy and breastfeeding and co-medication with a known CYP2D6 inhibitor. In addition, patients with a tamoxifen plasma concentration <100 nM at study baseline were excluded because this low plasma concentrations implied that these patients had missed at least one daily dose prior to sampling (Figure 2). The study design was as follows: Patients with EM/EM or EM/IM genotypes (activity score > 1) continued tamoxifen therapy at the standard dosage of 20 mg/day. Patients with EM/PM or IM/IM genotypes (activity score 1) received 30 mg/day (given as once 10 mg and once 20 mg) and patients with IM/PM or PM/PM genotypes (activity score 0.5 and 0, respectively) received 40 mg/day (given as 20 mg twice). Registration ID in IRCT was: IRCT2015082323734N1^2^.

During the follow-up period of 8 months plasma samples for the analysis of tamoxifen and its metabolites were drawn 4 and 8 months after inclusion of the patients. After 8 months the study was terminated. The decision whether treatment of patients with dose adjustment for tamoxifen should be continued was completely under the responsibility of the attending physician.

### Measuring the Concentration of Tamoxifen and Its Metabolites in Plasma

The plasma concentrations of tamoxifen and its metabolites were analyzed before and after 4 and 8 months of dose adjustment using LC-MS/MS as previously described (Mürdter et al., 2011).

In brief, sample preparation was carried out by protein precipitation and dilution. For calibration and method validation, calibration samples and quality controls were prepared by adding mixtures of all reference compounds in acetonitrile to blank plasma. Final concentrations of calibration samples were chosen according to the concentrations of metabolites expected in plasma of patients under steady state condition (for calibration ranges see Supplementary Table S1). For α-OH-DM-Tam no pure reference material was available; plasma concentrations were calculated using the calibration data of α-OH-Tam.

Chromatographic separation of tamoxifen and its metabolites was performed using a ZORBAX Eclipse plus C18 (Agilent Technologies, Waldbronn, Germany) and 1200 rapid resolution LC-system (Agilent) with a binary pump using a gradient of 0.1% formic acid in acetonitrile in 0.1% formic acid in water. A 6460 triple quadrupole mass spectrometer (Agilent) equipped with a Jet Stream electrospray source (Agilent) was used for MRM. MRM transitions, retention times and internal standards are given in the Supplementary Table S1.

### Toxicities Evaluation

Adverse events before and during the study were evaluated by questionnaire according to CTCAE v4.0. In addition, standard laboratory parameters such as ALT, AST, ALP, BUN, and creatinine were determined before and after 4 and 8 months.

### Statistical Analysis

For statistical analysis, SPSS software version 16.0 (SPSS, Inc., Chicago, IL, United States) was utilized. The differences of the plasma concentrations of tamoxifen and its metabolites between different genotypes were evaluated using Kruskal–Wallis test followed by Dunn’s *post hoc* test. The concentrations of tamoxifen and its metabolites before and after tamoxifen dose escalation were compared using Friedman’s test followed by Dunn’s multiple comparison test as a *post hoc* which compared 0 with 4, 0 with 8, and 4 with 8 months. The Friedman’s test was also applied to evaluate the associations between the increase of tamoxifen dosage and the incidence of adverse events. The results were considered statistically significant if the *p* value was less than or equal to 0.05.

## Results

### Allele and Genotype Frequencies and Assigned CYP2D6 Phenotypes

*CYP2D6* ^∗^1, ^∗^2, ^∗^4, ^∗^5, ^∗^6, ^∗^10, ^∗^17, and ^∗^41 alleles were successfully genotyped in 134 breast cancer patients. For copy number analysis 2 samples showed ambiguous results and therefore were excluded from further analysis (Figure 2). Allele and genotype frequencies of the study population are given in Supplementary Table S2. All genotypes were in Hardy–Weinberg equilibrium (*p* > 0.05).

Genotypes and the respective phenotypes based on the CYP2D6 activity score (AS) are shown in Table 2. The following phenotypes were assigned: UM (AS 3): ^∗^1/^∗^1, ^∗^1/^∗^2 or ^∗^2/^∗^2 and a copy number ≥ 3; EM/EM (AS: 2): ^∗^1/^∗^1, ^∗^1/^∗^2, ^∗^2/^∗^2, and ^∗^1/^∗^41 and ^∗^1/^∗^4 with copy number = 3; EM/IM (AS: 1.5): ^∗^1/^∗^10, ^∗^1/^∗^17, ^∗^1/^∗^41, and ^∗^2/^∗^41; EM/PM (AS: 1): ^∗^1/^∗^4; IM/IM (AS: 1): ^∗^10/^∗^10, ^∗^10/^∗^41, and ^∗^41/^∗^41; IM/PM (AS 0.5): ^∗^41/^∗^5; PM/PM (AS: 0): ^∗^4/^∗^4 and ^∗^4/^∗^6.

**Table 2:** *CYP2D6* phenotypes assigned according to the CYP2D6 activity scores and respective *CYP2D6* genotypes.

CYP2D6 phenotype			*CYP2D6*
(activity score)	*n*	(%)	genotype	*n*	(%)
UM (3)	11	(8.33)	^∗^1/^∗^1xN	2	(1.52)
			^∗^1/^∗^2xN	6	(4.55)
			^∗^2/^∗^2xN	3	(2.27)
EM (2)	68	(51.51)	^∗^1/^∗^1	16	(12.12)
			^∗^1/^∗^2	23	(17.42)
			^∗^2/^∗^2	26	(19.7)
			^∗^1/^∗^41xN	2	(1.52)
			^∗^1/^∗^4xN	1	(0.76)
EM/IM (1.5)	21	(15.90)	^∗^1/^∗^10	5	(3.79)
			^∗^1/^∗^17	1	(0.76)
			^∗^1/^∗^41	14	(10.61)
			^∗^2/^∗^41	1	(0.76)
EM/PM (1)	23	(17.42)	^∗^1/^∗^4	19	(14.39)
			^∗^1/^∗^5	3	(2.27)
			^∗^2/^∗^5	1	(0.76)
IM/IM (1)	4	(3.03)	^∗^10/^∗^10	1	(0.76)
			^∗^10/^∗^41	1	(0.76)
			^∗^41/^∗^41	2	(1.52)
IM/PM (0.5)	1	(0.76)	^∗^41/^∗^5	1	(0.76)
PM (0)	4	(3.03)	^∗^4/^∗^4	2	(1.52)
			^∗^4/^∗^5	1	(0.76)
			^∗^4/^∗^6	1	(0.76)

### Plasma Concentrations of Tamoxifen and Its Metabolites at Baseline

Steady-state plasma concentrations of tamoxifen and its metabolites before dose escalation were measured by LC-MS/MS (Table 3). The mean concentration of tamoxifen at baseline was 378 ± 176 nmol/l (*n* = 117). The most abundant metabolites of tamoxifen were N-desmethyl-tamoxifen (DM-Tam) whose concentration was approximately twice the concentration of tamoxifen followed by N,N-didesmethyltamoxifen (DDM-Tam), and (Z)-endoxifen. In contrast, the plasma concentrations of the other highly potent tamoxifen metabolite, (Z)-4-hydroxytamoxifen [(Z)-4-OH-Tam] was less than 1/5 of the concentration of (Z)-endoxifen. Of note, the less potent 3-hydroxylated metabolites 3-hydroxy-N-desmethyltamoxifen (3-OH-DM-Tam) and 3-hydroxytamoxifen (3-OH-Tam) showed only low concentrations compared to the respective 4-hydroxy-isomers. The concentrations of the (E)-isomers of both highly active metabolites, were highly variable and accounted for only 1/10 of that of (Z)-endoxifen and (Z)-4-OH-Tam, respectively. Interestingly, with respect to the glucuronides this difference in concentration was less pronounced (0.582 ± 0.377 nmol/l for (Z)-DM-Tam-4-O-Gluc vs. 1.9 ± 1.35 nmol/l for (E)-DM-Tam-4-O-Gluc) pointing to a more rapid glucuronidation of the (E)-isomer.

**Table 3:** Steady state plasma concentrations of tamoxifen and its metabolites in breast cancer patients receiving 20 mg/day tamoxifen (*n* = 117^∗^).

Compound	Mean ± SD (nM)
Tam	378 ± 176
Tam-NO	21.7 ± 10.7
DM-Tam	675 ± 312
DDM-Tam	120.5 ± 52.9
(Z)-Endoxifen	29.6 ± 17.1
(E)-Endoxifen	4.92 ± 6.4
(Z)-4’-OH-DM-Tam	15.1 ± 7.7
(E)-4’-OH-DM-Tam	1.62 ± 1.28
3-OH-DM-Tam	3.16 ± 2.14
α-OH-DM-Tam^//^	3.38 ± 1.84
(Z)-4-OH-Tam^#^	5.3 ± 3.14
(Z)-4’-OH-Tam^#^	5.83 ± 2.99
3-OH-Tam	1.015 ± 0.647
α-OH-Tam	1.352 ± 0.721
(Z)-OH-DDM-Tam^#^	4.04 ± 2.29
Tam-N-Gluc	0.919 ± 0.755
(Z)-DM-Tam-4-O-Gluc^§^	0.582 ± 0.377
(E)-DM-Tam-4-O-Gluc^$^	1.9 ± 1.35
(E)-Tam-4-O-Gluc^$^	0.551 ± 0.405

*^∗^Non-adherent patients with plasma Tam concentrations <100 nM were excluded. ^//^No reference compound available. Quantification was done using the calibration curve of α-OH-Tam. ^#^The respective (E)-isomers could be quantified only in a minority of samples at low concentrations. ^§^This glucuronide results from the conjugation of (E)-4-OH-DM-Tam. ^$^These glucuronides result from conjugation of (Z)-endoxifen and (Z)-4-OH-Tam, respectively.*

### Relationship Between *CYP2D6* Genotypes and Plasma Metabolite Concentrations

**FIGURE 3:**
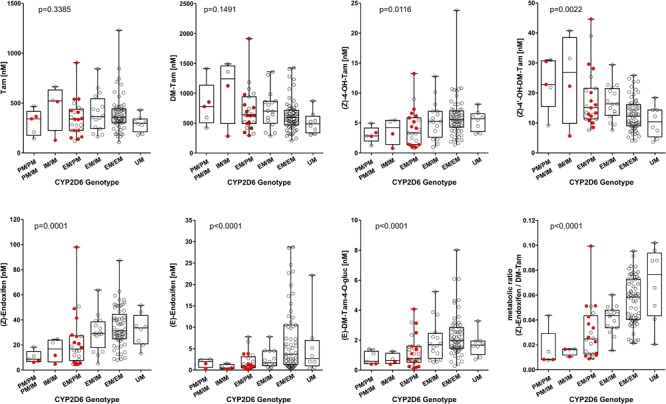
Plasma concentrations of selected tamoxifen metabolites stratified according to *CYP2D6* genotype in 117 Iranian breast cancer patients receiving 20 mg/day tamoxifen for at least 4 months. Non-adherent patients with plasma tamoxifen concentrations <100 nM were excluded. The patients who were scheduled for the dose escalation trial are depicted in red. The differences between the genotype groups were assessed by Kruskal–Wallis Test.

As CYP2D6 mediates a major step in the bioactivation of tamoxifen, we investigated the relationship between *CYP2D6* genotypes and the concentrations of tamoxifen and its metabolites (Figure 3 and Supplementary Table S3). Using Kruskal–Wallis test, the mean concentrations of (Z)-endoxifen, (E)-endoxifen and their respective glucuronides, (Z)-4-OH-Tam, 3-OH-DM-Tam, and 3-OH-Tam were significantly different between genotype groups (*p* < 0.05). Despite considerable inter-individual variability within the genotype groups, all these metabolites showed increasing plasma concentrations with increasing CYP2D6 activity. However, 4′-hydroxy-N-desmethyltamoxifen (4′-OH-DM-Tam) showed an inverse correlation with lower plasma concentrations in patients with higher CYP2D6 activity (*p* = 0.0022). On the other hand, there was no significant difference in plasma concentrations of tamoxifen, tamoxifen-N-oxide, DM-Tam, DDM-Tam, α-hydroxytamoxifen (a-OH-Tam) and α-hydroxy-N-desmethyltamoxifen (a-OH-DM-Tam). As (Z)-endoxifen can undergo chemical or enzymatic isomerization into (E)-endoxifen we also analyzed the sum of both isomers revealing an even better correlation with CYP2D6 activity (*p* < 0.0001). As a surrogate parameter for the intrinsic enzyme activity the MR of (Z)-endoxifen to DM-Tam correlates very well with CYP2D6 activity (*p* < 0.0001).

### Concentration of Tamoxifen and Its Metabolites Following Dose Adjustment

**FIGURE 4:**
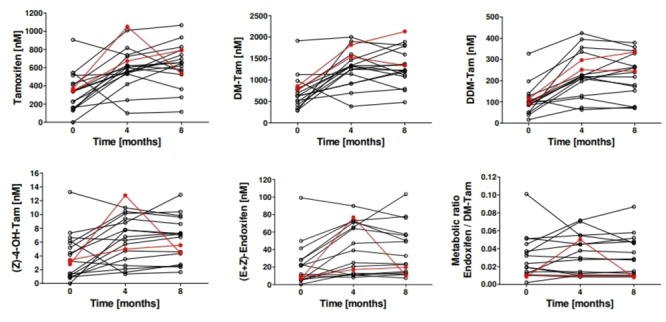
Plasma concentrations of tamoxifen and its main metabolites and the MR (Z)-endoxifen/DM-Tam in patients (*n* = 17) before and after dose escalation. Patients genotyped for *CYP2D6* as intermediate metabolizer (*n* = 15) (EM/PM or IM/IM) received 30 mg/day of tamoxifen and patients genotyped as poor metabolizers (*n* = 2) (PM/PM, IM/PM) received 40 mg/day. Plasma samples were drawn 4 and 8 months after dose adjustment and tamoxifen and its metabolites were quantified by UHPLC-MS/MS. The two patients who received 40 mg tamoxifen are shown in red.

Dose adjustment in patients with an activity score of 0–0.5 (*n* = 2) and 1 (*n* = 15) to 40 and 30 mg/day, respectively, resulted in a significant increase in median plasma concentrations of tamoxifen and all metabolites except the (E)-isomers of endoxifen and 4′-OH-DM-Tam and (E)-Tam-4-O-Gluc (Supplementary Table S4). After 8 months the median plasma concentrations increased by about 26–122% compared to baseline. Plasma concentrations of tamoxifen and its main metabolites and the MR (Z)-endoxifen/DM-Tam during dose escalation are shown in Figure 4. Due to the unexplained high variability of isomerization the concentrations of the isomers of endoxifen were additionally evaluated in sum. Of note, the MR endoxifen/DM-Tam did not change upon dose escalation. In line with this data, the increased intake of tamoxifen did not change the MR of other reactions involved in the formation or clearance of the anti-estrogenic metabolites (Z)-4-OH-Tam and (Z)-endoxifen.

### Adherence to Therapy

Among the 17 patients who completed the dose adjustment protocol compliance of study medication has been considered entirely satisfactory: 47.1% (*n* = 8) did not miss any tamoxifen dose, 29.4% (*n* = 5) and 17.6% (*n* = 3) missed 1–5 and 6–10 doses, respectively. One patient reported more than 10 missing tamoxifen doses.

### Toxicities and Adverse Events

No woman was withdrawn from the study due to metastasis, thromboembolic or other serious adverse events. Vomiting, nausea, sexual pain, bloating, and irritability were significantly more frequent in the patients under tamoxifen dose escalation (Table 4; Friedman test, *P* < 0.05). No changes in ALT, AST, ALP, BUN, and creatinine levels have been observed upon dose escalation.

**Table 4:** Side effects are shown in patients without dose escalation (*n* = 117) and for dose adjusted patients (*n* = 17) before, after 4 and 8 months of dose adjustment.

Test Side effects	Tamoxifen dosage	Time point	Number of patients reporting				Friedman *p*-value
			0	1	2	3	
Hot flashes	20 mg/day (*n* = 117)		13	34	40	30	
	Dose escalation	Baseline	2	4	6	5	0.439
	group (*n* = 15)	4 months	3	5	5	4	
		8 months	5	2	4	6	
Cold sweat	20 mg/day (*n* = 117)		58	20	22	17	
	Dose escalation	Baseline	9	3	3	2	0.358
	group (*n* = 15)	4 months	8	6	1	2	
		8 months	6	4	4	3	
Night sweat	20 mg/day (*n* = 117)		44	27	25	21	
	Dose escalation	Baseline	7	4	3	3	0.509
	group (*n* = 15)	4 months	6	8	2	1	
		8 months	6	4	4	3	
Vaginal irritation	20 mg/day (*n* = 117)		72	22	21	2	
	Dose escalation	Baseline	10	3	4	0	0.152
	group (*n* = 15)	4 months	7	3	4	3	
		8 months	6	3	2	6	
Vaginal bleeding	20 mg/day (*n* = 117)		102	3	8	4	
	Dose escalation	Baseline	15	0	1	1	0.662
	group (*n* = 15)	4 months	13	1	3	0	
		8 months	13	1	1	2	
Vaginal dryness	20 mg/day (*n* = 117)		55	44	17	1	
	Dose escalation	Baseline	7	7	3	0	0.549
	group (*n* = 15)	4 months	7	4	4	2	
		8 months	7	3	3	4	
Sexual unwillingness	20 mg/day (*n* = 117)		44	26	26	21	
	Dose escalation	Baseline	6	3	4	4	0.692
	group (*n* = 15)	4 months	7	3	4	3	
		8 months	6	3	5	3	
Sexual pain	20 mg/day (*n* = 117)		58	44	9	6	
	Dose escalation	Baseline	8	8	0	1	0.002
	group (*n* = 15)	4 months	6	5	2	4	
		8 months	6	3	4	4	
Weight gain	20 mg/day (*n* = 117)		53	34	12	18	
	Dose escalation	Baseline	8	4	2	3	0.309
	group (*n* = 15)	4 months	6	5	2	4	
		8 months	7	4	3	3	
Dizziness	20 mg/day (*n* = 117)		54	53	4	6	
	Dose escalation	Baseline	8	8	0	1	0.727
	group (*n* = 15)	4 months	7	7	0	3	
		8 months	8	6	0	3	
Vomiting	20 mg/day (*n* = 117)		101	12	4	0	
	Dose escalation	Baseline	15	2	0	0	0.009
	group (*n* = 15)	4 months	12	4	1	0	
		8 months	10	6	1	0	
Diarrhea	20 mg/day (*n* = 117)		104	13	0	0	
	Dose escalation	Baseline	15	2	0	0	0.819
	group (*n* = 15)	4 months	14	3	0	0	
		8 months	14	2	0	1	
Bloating	20 mg/day (*n* = 117)		46	34	22	15	0.023
	Dose escalation	Baseline	7	4	3	3	
	group (*n* = 15)	4 months	4	3	6	4
		8 months	5	4	5	3
Mood swing	20 mg/day (*n* = 117)		14	66	26	11	
	Dose escalation	Baseline	2	9	4	2	0.423
	group (*n* = 15)	4 months	3	4	7	3	
		8 months	4	4	6	3	
Irritability	20 mg/day (*n* = 117)		24	50	31	12	
	Dose escalation	Baseline	3	7	5	2	0.006
	group (*n* = 15)	4 months	3	2	6	6	
		8 months	4	1	7	5	

## Discussion

There is increasing evidence that personalized therapy in oncology contributes significantly to better treatment outcome with a tolerable safety (Meyer et al., 2013; Relling and Evans, 2015). Recently, genotyping of *CYP2D6* in patients undergoing tamoxifen therapy for premenopausal and postmenopausal breast cancer patients has been proposed to improve treatment outcome (Province et al., 2014;Goetz et al., 2018). Previous clinical trials in breast cancer showed first evidence that *CYP2D6* dose-adjusted treatment of tamoxifen resulted in significantly increased plasma concentrations of endoxifen, the major active metabolite of tamoxifen (Barginear et al., 2011; Irvin et al., 2011; Kiyotani et al., 2012; de Duenas et al., 2014; Dezentjé et al., 2015; Fox et al., 2016; Hertz et al., 2016).

To the best of our knowledge currently no data are available demonstrating the feasibility of individualized *CYP2D6* guided tamoxifen therapy in an Iranian population of breast cancer patients. Therefore, we initiated a prospective clinical pilot study to adjust tamoxifen dosage in ER^+^ breast cancer patients with Iranian ethnic background. Considering major differences of *CYP2D6* allele frequencies world-wide (Zanger and Schwab, 2013), genotyping of *CYP2D6* was performed covering *CYP2D6* alleles relevant for the Middle East population (Sistonen et al., 2007; Bagheri et al., 2015). Based on our study protocol and in agreement with previous studies elucidating the impact of *CYP2D6*-guided tamoxifen dose adjustment in patients with compromised CYP2D6 activity (PM and IM), plasma concentrations of tamoxifen and selected metabolites were quantified before, after 4 and 8 months of dose escalation. In addition to DM-Tam, (Z)-endoxifen, and 4-OH-Tam which are suggested to be clinically relevant, we comprehensively measured other tamoxifen metabolites by a previously established and validated sensitive and highly specific LC-MS/MS method (Mürdter et al., 2011). This method includes various hydroxylated metabolites and glucuronides (Figure 1; Brauch et al., 2009).

At baseline, the inter-individual variability of tamoxifen and selected metabolites in the Iranian population was similar to data in other ethnic populations of breast cancer patients (e.g., Europe, America and Asians) (Saladores et al., 2015). Regarding (Z)-endoxifen, our data confirms the close relationship between *CYP2D6* genotypes and plasma concentrations proposed by other studies (Lim et al., 2011; Antunes et al., 2015; Schroth et al., 2017; Woo et al., 2017). Due to the small number of patients who were enrolled in our pilot study to evaluate dose escalation of tamoxifen in Iranian breast cancer, data analyses have been performed by combining IM and PM patients treated with 30 and 40 mg tamoxifen per day, respectively. Generally our data demonstrate a significant increase in plasma concentrations of tamoxifen and its metabolites following dose escalation for 4 and 8 months. Of note, (Z)-endoxifen plasma concentrations in patients with compromised CYP2D6 activity and dose escalation were similar to those in patients with CYP2D6 EM phenotype at standard dosage.

These data are in line with previous studies demonstrating that dose escalation of tamoxifen in CYP2D6 compromised breast cancer patients result in higher plasma levels of the active metabolite endoxifen. In the study by Irvin et al. (2011) in a US American population the concentration of endoxifen was increased in both, the IM and PM patients, after doubling the tamoxifen dosage to 40 mg/day for 4 months. The endoxifen plasma levels in the IM group receiving 40 mg/day tamoxifen were similar to the EM group treated with 20 mg/day (21.8 vs. 29.2 ng/ml; *p* = 0.84). In contrast, patients in the PM group failed to reach similar endoxifen plasma levels (12.9 vs. 29.2 ng/ml; *p* = 0.016) despite dose escalation. Barginear et al. (2011) increased the tamoxifen dosage from 20 to 30 mg/day based on plasma endoxifen concentration (<40 nmol/l) in US patients with various ethnic background and IM CYP2D6 phenotype. Dose adjustment resulted in elevated levels of (Z)-endoxifen, (Z)-4′-OH-DM-Tam, (Z)-4-OH-Tam, and (Z)-4′-OH-Tam. Noteworthy, in line with our data lower plasma concentrations of (Z)-4′-OH-DM-Tam in patients with an EM CYP2D6 activity score compared to IM/PM patients were reported, indicating a metabolic shift from (Z)-4′-OH-DM-Tam to endoxifen. In an Asian population Kiyotani et al. (2012) investigated dose escalation of tamoxifen to 30 and 40 mg/day in patients who were heterozygous and homozygous for *CYP2D6* IM alleles, respectively. After 8 weeks, concentrations of tamoxifen, N-DM-Tam, 4-OH-Tam and endoxifen were increased in IM patients resulting in endoxifen and 4-OH-Tam levels similar to those detected in EM patients. After enrollment of the patients in the CYPTAM study, tamoxifen dose escalation for IM and PM patients was calculated by the formula: 20 mg × (average endoxifen serum concentration in EMs divided by the patient’s baseline endoxifen serum concentration) and led to tamoxifen doses between 30–100 mg for the IMs and 60–120 mg for PMs (Dezentjé et al., 2015). Dose adjustment resulted in elevated levels of endoxifen in both PMs and IMs. There was no significant difference with the mean endoxifen level in EMs (=33.7 nM), and IMs after dose escalation (=30.3 nM; *p* = 0.20), although the mean endoxifen level in PMs after dose escalation was significantly lower (=27.3 nM; *p* = 0.03).

Building on the success of the pilot study by Irvin et al. (2011) and Hertz et al. (2016) expanded enrollment to 500 patients and again they doubled the tamoxifen dosage from 20 to 40 mg/day in both, the IM and PM patients. After 4 months, the endoxifen plasma levels in the genotype-dosed IM patients, had risen by 48% to 10.74 ng/mL, which was no longer different from, and was in fact nominally greater than, the endoxifen concentrations in EM/UM patients (9.30 ng/mL, *p* = 0.08). Endoxifen concentrations in genotype-dosed PM patients also rose by approximately 61% to 5.52 ng/mL but remained significantly lower than in EM/ UM patients (9.30 ng/ml; *p* = 0.009). In the Australian TADE study patients with basal endoxifen concentrations <30 nmol/l underwent a sequential dose escalation scheme with a maximum tamoxifen dose of 60 mg/day resulting in endoxifen concentrations >30 nmol/l in 76% (93 of 122) of participants (Fox et al., 2016). Of note patients with a PM phenotype, i.e., carrying two non-functional *CYP2D6* alleles were unable to achieve the target endoxifen concentration of 30 nmol/l despite dose escalation.

Independent from *CYP2D6* genotype increased dosing of tamoxifen to 30 and 40 mg/day resulted in significantly higher plasma levels of tamoxifen compared to tamoxifen standard treatment (20 mg/day).

Since altered pharmacokinetics may result in a higher rate of ADR, we systematically monitored tamoxifen specific side effects as well as laboratory parameters indicating liver and kidney toxicity. No severe side effects (e.g., thromboembolism) occurred during dose escalation. Moreover there was no significant increase in the occurrence of hot flashes and only slightly increased frequencies of vomiting and agitation. These data corroborate previous studies demonstrating that doses escalation of tamoxifen in CYP2D6 compromised breast cancer patients is well tolerated (Barginear et al., 2011; Irvin et al., 2011; Kiyotani et al., 2012; Dezentjé et al., 2015; Fox et al., 2016; Hertz et al., 2016).

Besides CYP2D6, CYP2C9, and CYP2C19 as well as some phase 2 enzymes have also been reported to participate in tamoxifen pharmacokinetics, however, with far less impact than CYP2D6 (Mürdter et al., 2011; Saladores et al., 2015; Marcath et al., 2017; Sutiman et al., 2016). Therefore, this study was focused on CYP2D6.

A limitation of our study is the number of patients included. Previous studies have shown a low frequency of CYP2D6 PM subjects in the Iranian population (Kouhi et al., 2009; Hashemi-Soteh et al., 2011; Bagheri et al., 2015). In line with these findings, the number of PM patients was not high enough to analyze those patients separately. Moreover no outcome data are available.

Taken together, for the first time we showed that the concept of dose escalation of tamoxifen in CYP2D6 compromised breast cancer patients is feasible in an Iranian population resulting in significantly higher plasma concentrations of the active metabolite (Z)-endoxifen. Our study supports ongoing and future activities to comprehensively elucidate the impact of *CYP2D6*- guided dose escalation on clinical outcome in prospective clinical trials. The strategy of dose escalation in patients with compromised genetics for drug metabolizing enzymes regarding other drugs as tamoxifen appears to be a promising approach which warrants future trials.

## Ethics Statement

This study was carried out in accordance with the recommendations of the Ethical Committee of Isfahan University of Medical Sciences with written informed consent from all subjects. All subjects gave written informed consent in accordance with the Declaration of Helsinki. The protocol was approved by the Ethical Committee of Isfahan University of Medical Sciences.

## Author Contributions

ZK, ZB, PN, HK, RS, and ManS designed the project and involved in doing the experiments. MatS and TM performed the measurements of tamoxifen metabolite concentrations. ES performed the *CYP2D6* CNV analysis. ZK, ZB, MatS, TM, ManS, and PN analyzed the data and wrote the manuscript. FM was the clinician of the project, who helped us in designing the project, sample collection, and data analysis. All authors read and approved the final manuscript.

## Conflict of Interest Statement

The authors declare that the research was conducted in the absence of any commercial or financial relationships that could be construed as a potential conflict of interest.

## Footnotes

1 https://www.cog-genomics.org/software/stats

2 https://www.irct.ir/trial/20231

## Abbreviations

ADR: adverse drug reaction
ALP: alkaline phosphatase
ALT: Alanine transaminase
AST: Aspartate transaminase
BUN: blood urea nitrogen
CTCAE v4.0: Common Toxicity Criteria for Adverse Events, version 4
CYP2D6: cytochrome P450 2D6
DDM: N, N-DiDesmethyl
DM: N-desmethyl
EM: extensive metabolizer
ER: estrogen receptor
Gluc: Glucuronides
IM: intermediate metabolizer
IRCT: Iranian Registry of Clinical Trials
LC-MS/MS: liquid chromatography tandem mass spectrometry
MR: metabolic ratio
MRM: multiple reaction monitoring
OH: hydroxyl
PM: poor metabolizer
SPSS: Statistical Package for Social Sciences
Tam: tamoxifen
UM: ultra-rapid metabolizer
