# Supplementary material for: Clinical Trial: CYP2D6 Related Dose Escalation of Tamoxifen in Breast Cancer Patients With Iranian Ethnic Background Resulted in Increased Concentrations of Tamoxifen and Its Metabolites
Source: Front Pharmacol. 2019 May 24;10:530. doi: 10.3389/fphar.2019.00530 (PMC6543868; doi:10.3389/fphar.2019.00530)
Supplement: Supplementary file 1 [file Table_1.pdf]

Supplementary Data:

Supplementary Table S1: UHPLC-MS/MS Parameter

**Compound**	**MS/MS Transition**	**RT [min]**	**Linear range [nM]**	**ISTD Compound**	**ISTD Conc.**
α-OH-DM-Tam	374.2 -> 58.1	3.90	see of a-OH-Tam[#]	4-OH-Tam d5 E	7.8
DM-Tam-4-O-Gluc E	550.3 -> 58.1	3.95	9.00 – 0.15	Tam-4-O-Gluc d5 E	9
α-OH-Tam	388.2 -> 72.1	4.07	3.00 – 0.05	4-OH-Tam d5 E	7.8
Tam-4-O-Gluc E	564.3 -> 72.1	4.15	5.64 – 0.094	Tam-4-O-Gluc d5 E	9
DM-Tam-4-O-Gluc Z	550.3 -> 58.1	4.20	6.00 – 0.10	Tam-4-O-Gluc d5 Z	11
4'-OH-DM-Tam E	374.2 -> 58.1	4.65	0.60 – 0.02	Endoxifen d5 E	19.2
Endoxifen E	374.2 -> 58.1	7.00	15.0 – 0.25	Endoxifen d5 E	19.2
OH-DDM-Tam Z	360.2 -> 44.1	7.10	23.2 – 0.39	Endoxifen d5 Z	20.8
Endoxifen Z	374.2 -> 58.1	7.50	150 – 2.5	Endoxifen d5 Z	20.8
4-OH-Tam Z	388.2 -> 72.1	7.90	17.4 – 0.29	4-OH-Tam d5 Z	12.2
3-OH-DM-Tam	374.2 -> 58.1	7.90	9.00 – 0.15	Endoxifen d5 Z	20.8
3-OH-Tam	388.2 -> 72.1	8.30	3.00 – 0.05	4-OH-Tam d5 Z	12.2
4'-OH-DM-Tam Z	374.2 -> 58.1	8.90	60.0 – 1.0	Endoxifen d5 Z	20.8
4'-OH-Tam Z	388.2 -> 72.1	9.30	29.4 – 0.49	4-OH-Tam d5 Z	12.2
DDM-Tam	344.2 -> 44.1	10.40	300 – 5.0	DM-Tam d5	400
Tam-N-Gluc	548.3 -> 372.1	10.50	18.0 – 0.30	Tam d3	200
DM-Tam	358.2 -> 58.1	10.60	2,100 – 35.0	DM-Tam d5	400
Tam	372.2 -> 72.1	10.70	1,500 – 25.0	Tam d3	200
Tam-NO	388.2 -> 72.1	10.85	150 – 2.5	DM-Tam d5	400

**ISTD Compound**	**Transition**	**RT [min]**
Tam-4-O-Gluc d5 E	569.3 -> 72.1	4.17
Tam-4-O-Gluc d5 Z	569.3 -> 72.1	4.40
Endoxifen d5 E	379.2 -> 58.1	7.00
4-OH-Tam d5 E	393.2 -> 72.1	7.40
Endoxifen d5 Z	379.2 -> 58.1	7.50
4-OH-Tam d5 Z	393.2 -> 72.1	7.90
DM-Tam d5	363.2 -> 58.1	10.50
Tam d3	375.2 -> 75.1	10.70

[#]: no reference compound available, for quantification of α-OH-DM-Tam calibration data of α-OH-Tam was used.

Supplementary table S2: *CYP2D6* alleles, variants, polymorphisms and observed Allele frequencies of 132 Patients

***CYP2D6* allele**	**Variant**	**SNP**	**Genotypes**			***P**
			Homozygous (Wt/Wt)	Heterozygous (Wt/V)	Homozygous (V/V)	
CYP2D6 *2	4180G>C	rs1135840	22	74	36	0.16
CYP2D6 *4	1846 G>A	rs3892097	108	22	2	0.36
CYP2D6 *5	Gene del	-	126	6	0	1
CYP2D6 *6	1707 T del	rs5030655	131	1	0	1
CYP2D6 *10	100C>T	rs1065852	101	28	3	0.45
CYP2D6 *17	1023C>T	rs38271706	131	1	0	1
CYP2D6 *41	2988G>A	rs28371725	111	19	2	0.25
-	Gene dup	-	118	14	0	1

Abbreviations: Wt: wild type, V: variant, del: deletion, dup: duplication.

**P* value of the exact test on deviation from the Hardy-Weinberg equilibrium

Supplementary Table S3: Plasma concentrations of tamoxifen and its metabolites in different genotype groups

**Metabolite [Nm]**	**Total**	**UM**	**EM/EM**	**EM/IM**	**EM/PM**	**IM/IM**	**PM/PM IM/PM**	***P***	**Significant differences following Dunn's post hoc test**
**Tam**$_{\#}$<br>Median<br>(min, max)<br>n	346.891<br>(40.71, 1231.702)<br>123	284.349<br>(58.199, 431.250)<br>10	355.641<br>(40.71, 1231.702)<br>64	359.326<br>(90.711, 844.138)<br>17	339.376<br>(120.643, 906.167)<br>23	522.243<br>(129.165, 665.678)<br>4	345.556<br>(145.243, 471.260)<br>5	0.338	-
**Tam-NO**<br>Median<br>(min, max)<br>n	19.585<br>(5.12, 67.47)<br>121	15.721<br>(9.002, 35.575)<br>9	19.858<br>(5.490, 63.758)<br>63	21.457<br>(5.979, 67.474)<br>17	19.396<br>(5.582, 35.944)<br>23	26.586<br>(11.514, 40.360)<br>4	21.084<br>(5.123, 28.622)<br>5	0.447	-
**DM-Tam**<br>Median<br>(min, max)<br>n	613.758<br>(80.92, 1913.33)<br>122	424.168<br>(86.280, 871.113)<br>10	580.112<br>(80.924, 1424.820)<br>63	694.451<br>(142.154, 1360.602)<br>17	638.023<br>(293.867, 1913.330)<br>23	1241.635<br>(282.931, 1490.261)<br>4	777.908<br>(419.660, 1415.642)<br>5	0.149	-
**DDM-Tam**<br>Median<br>(min, max)<br>n	110.694<br>(11.77, 327.28)<br>123	98.529<br>(32.252, 140.507)<br>10	123.683<br>(11.769, 249.979)<br>64	110.069<br>(19.595, 228.394)<br>17	98.772<br>(16.329, 327. 278)<br>23	146.488<br>(50.151, 214.202)<br>4	91.275<br>(69.946, 195.529)<br>5	0.618	-
**Endoxifen Z**<br>Median<br>(min, max)<br>n	27.505<br>(4.5, 97.92)<br>121	27.970<br>(8.665, 51.526)<br>10	30.602<br>(5.864, 87.302)<br>63	28.864<br>(5.154, 63.758)<br>17	16.804<br>(4.505, 97.923)<br>22	16.803<br>(4.740, 24.579)<br>4	8.621<br>(6.289, 18.264)<br>5	<0.001	EM/EM with EM/PM<br>EM/EM with PM/PM
**Endoxifen E**<br>Median<br>(min, max)<br>n	2.264<br>(0.21, 28.75)<br>115	2.678<br>(0.354, 22.164)<br>10	3.697<br>(0.247, 28.747)<br>61	1.421<br>(0.379, 7.792)<br>15	0.936<br>(0.221, 7.784)<br>21	0.522<br>(0.213, 1.564)<br>4	1.955<br>(0.309, 2.534)<br>4	<0.0001	EM/EM EM/PM
**Endoxifen E+Z**<br>Median<br>(min, max)<br>n	31.385<br>(0.44, 99.57)<br>122	35.168<br>(9.644, 52.162)<br>10	38.260<br>(6.476, 99.574)<br>63	30.759<br>(6.423, 68.343)<br>17	21.563<br>(0.443, 99.186)<br>23	17.691<br>(5.247, 25.115)<br>4	8.629<br>(6.598, 20.661)<br>5	<0.001	EM/EM with EM/PM<br>EM/EM with PM/PM
**4-OH-Tam Z**<br>Median<br>(min, max)<br>n	5.179<br>(0.81, 23.81)<br>122	5.005<br>(1.291, 8.149)<br>10	5.334<br>(0.934, 23.814)<br>63	5.254<br>(0.981, 12.781)<br>17	3.345<br>(0.846, 13.244)<br>23	4.214<br>(0.812, 5.504)<br>4	2.846<br>(1.209, 4.939)<br>5	0.012	-
**3-OH-DM-Tam**<br>Median<br>(min, max)<br>n	2.767<br>(0.24, 10.83)<br>122	3.128<br>(0.853, 4.756)<br>10	3.234<br>(0.367, 10.826)<br>63	2.766<br>(0.564, 5.402)<br>17	1.541<br>(0.245, 9.429)<br>23	1.711<br>(0.342, 3.378)<br>4	1.137<br>(0.618, 2.440)<br>5	<0.001	EM/EM with EM/PM<br>EM/EM with PM/PM
**3'-OH-DM-Tam**<br>Median<br>(min, max)<br>n	2.686<br>(1.87, 6.65)<br>95	2.345<br>(1.873, 3.070)<br>6	2.689<br>(1.879, 6.647)<br>52	2.865<br>(1.985, 3.995)<br>14	2.636<br>(1.955, 5.308)<br>16	3.923<br>(2.232, 4.666)<br>3	2.494<br>(2.291, 4.123)<br>4	0.620	-
**4'-OH-DM-Tam Z**<br>Median<br>(min, max)<br>n	13.042<br>(2.16, 44.60)<br>123	8.009<br>(2.431, 18.444)<br>10	11.360<br>(2.157, 25.877)<br>64	16.308<br>(4.191, 29.402)<br>17	15.055<br>(7.583, 44.604)<br>23	26.870<br>(5.662, 40.750)<br>4	22.820<br>(9.147, 31.018)<br>5	0.002	UM with EM/PM<br>UM with PM/PM

**4'-OH-DM-Tam E**<br>Median<br>(min, max)<br>n	1.271<br>(0.1, 7.59)<br>122	1.078<br>(0.101, 4.138)<br>10	1.207<br>(0.113, 4.884)<br>63	1.119<br>(0.154, 2.643)<br>17	1.617<br>(0.185, 3.952)<br>23	0.880<br>(0.663, 1.663)<br>4	1.846<br>(0.276, 7.590)<br>5	0.663	-
**3-OH-Tam**<br>Median<br>(min, max)<br>n	0.880<br>(0.1, 3.19)<br>122	0.849<br>(0.286, 1.537)<br>10	0.972<br>(0.138, 3.186)<br>63	0.954<br>(0.164, 1.870)<br>17	0.652<br>(0.129, 2.681)<br>23	0.730<br>(0.201, 1.488)<br>4	0.579<br>(0.100, 1.286)<br>5	0.039	EM/EM with EM/PM
**3'-OH-Tam**<br>Median<br>(min, max)<br>n	1.266<br>(0.80, 2.89)<br>92	1.015<br>(0.818, 1.417)<br>4	1.286<br>(0.803, 2.886)<br>53	1.103<br>(0.840, 1.653)<br>14	1.199<br>(0.806, 2.606)<br>14	1.794<br>(1.227, 1.920)<br>3	1.286<br>(1.185, 1.721)<br>4	0.394	-
**4'-OH-Tam Z**<br>Median<br>(min, max)<br>n	5.191<br>(1.29, 18.20)<br>122	3.873<br>(1.287, 7.266)<br>10	5.272<br>(1.421, 18.202)<br>63	5.766<br>(1.807, 11.471)<br>17	4.894<br>(2.474, 14.909)<br>23	8.101<br>(2.295, 13.884)<br>4	5.277<br>(3.726, 7.482)<br>5	0.279	-
**a-OH-DM-Tam**<br>Median<br>(min, max)<br>n	2.988<br>(0.09, 16.07)<br>123	2.137<br>(0.576, 4.106)<br>10	3.202<br>(0.092, 6.974)<br>64	3.481<br>(0.712, 4.907)<br>17	2.869<br>(1.358, 16.073)<br>23	5.073<br>(0.831, 5.195)<br>4	4.002<br>(1.546, 5.096)<br>5	0.193	-
**a-OH-Tam**<br>Median<br>(min, max)<br>n	1.267<br>(0.17, 5.60)<br>122	0.934<br>(0.248, 1.328)<br>10	1.314<br>(0.170, 3.286)<br>63	1.361<br>(0.227, 1.932)<br>17	1.047<br>(0.419, 5.604)<br>23	1.859<br>(0.287, 2.028)<br>4	1.352<br>(0.357, 1.591)<br>5	0.120	-
**OH-DDM-Tam Z**<br>Median<br>(min, max)<br>n	3.522<br>(0.64, 12.36)<br>122	3.361<br>(0.948, 7.430)<br>10	3.505<br>(0.642, 12.348)<br>64	3.733<br>(0.825, 11.834)<br>17	3.224<br>(0.748, 12.365)<br>22	4.432<br>(0.799, 6.301)<br>4	3.524<br>(2.397, 5.953)<br>5	0.973	-
**Tam-N-Gluc**<br>Median<br>(min, max)<br>n	0.670<br>(0.25, 3.91)<br>116	0.456<br>(0.250, 3.667)<br>8	0.683<br>(0.278, 3.909)<br>61	0.496<br>(0.346, 3.093)<br>16	0.755<br>(0.287, 2.935)<br>22	0.963<br>(0.434, 1.198)<br>4	0.638<br>(0.432, 0.766)<br>5	0.350	-
**DM-Tam-4-O-Gluc E**<br>Median<br>(min, max)<br>n	1.566<br>(0.13, 8.01<br>123	1.667<br>(0.718, 3.281)<br>10	1.925<br>(0.134, 8.014)<br>64	1.631<br>(0.254, 5.234)<br>17	0.791<br>(0.132, 4.071)<br>23	0.646<br>(0.403, 1.254)<br>4	0.577<br>(0.412, 1.405)<br>5	<0.0001	EM/EM with EM/PM
**DM-Tam-4-O-Gluc Z**<br>Median<br>(min, max)<br>n	0.453<br>(0.16, 2.04)<br>112	0.433<br>(0.210, 1.407)<br>10	0.538<br>(0.161, 2.043)<br>61	0.496<br>(0.181, 1.572)<br>16	0.376<br>(0.165, 1.221)<br>18	0.306<br>(0.177, 0.374)<br>3	0.287<br>(0.180, 0.402)<br>4	0.039	-
**Tam-4-O-Gluc E**<br>Median<br>(min, max)<br>n	0.476<br>(0.16, 3.25)<br>111	0.424<br>(0.171, 0.719)<br>9	0.512<br>(0.181, 3.247)<br>61	0.445<br>(0.163, 1.464)<br>15	0.406<br>(0.175, 0.980)<br>18	0.321<br>(0.180, 0.482)<br>4	0.286<br>(0.228, 0.646)<br>4	0.189	-
**DM-Tam-3-O-Gluc**<br>Median<br>(min, max)<br>n	4.092<br>(0.22, 22.9)<br>123	3.267<br>(1.487, 9.182)<br>10	4.828<br>(0.225, 22.898)<br>64	3.550<br>(0.573, 14.463)<br>17	2.727<br>(0.300, 11.601)<br>23	2.694<br>(1.124, 4.866)<br>4	1.601<br>(1.111, 4.449)<br>5	0.003	EM/EM with EM/PM
**DM-Tam-3'-O-Gluc**<br>Median<br>(min, max)	1.986<br>(0.37, 10.65)	1.370<br>(0.598, 6.241)	1.981<br>(0.663, 10.646)	2.090<br>(0.366, 7.509)	2.098<br>(0.430, 6.717)	2.796<br>(1.178, 3.206)	1.780<br>(1.041, 5.858)	0.825	-

n	121	10	62	17	23	4	5		
**Tam-3-O-Gluc**<br>Median<br>(min, max)<br>n	0.744<br>(0.08, 4.35)<br>122	0.595<br>(0.224, 1.546)<br>10	0.872<br>(0.075, 4.351)<br>64	0.753<br>(0.135, 3.195)<br>17	0.629<br>(0.077, 2.328)<br>22	0.754<br>(0.289, 1.143)<br>4	0.502<br>(0.118, 1.458)<br>5	0.100	-
**Tam-3'-O-Gluc**<br>Median<br>(min, max)<br>n	1.144<br>(0.19, 6.03)<br>122	0.791<br>(0.235, 4.531)<br>10	1.166<br>(0.332, 6.030)<br>63	1.016<br>(0.212, 4.256)<br>17	1.085<br>(0.194, 3.311)<br>23	1.390<br>(0.730, 2.060)<br>4	1.107<br>(0.267, 3.742)<br>5	0.831	-
**3-OH-4-Ome-Tam**<br>Median<br>(min, max)<br>n	0.321<br>(0.20, 1.91)<br>71	0.309<br>(0.234, 0.590)<br>5	0.305<br>(0.200, 1.907)<br>37	0.338<br>(0.207, 0.531)<br>11	0.383<br>(0.233, 1.097)<br>12	0.512<br>(0.451, 0.574)<br>2	0.333<br>(0.254, 0.457)<br>4	0.646	-

**P*: *P* value of Kruskal-Wallis test

Supplementary Table S4: Plasma concentrations of tamoxifen and its metabolites in patients before and after dose adjustment. Initially, all patients were treated with 20 mg/d tamoxifen. Patients genotyped for *CYP2D6* as intermediate metabolizer (EM/PM or IM/IM; n=15) received 30 mg/d of tamoxifen and patients genotyped as poor metabolizers (PM/PM, IM/PM; n=2) received 40 mg/d. Plasma samples were drawn 4 and 8 months after dose adjustment and tamoxifen and its metabolites were quantified by UHPLC-MS/MS

Compound	time of dose adjustment [Months]	median [nM]	(range) [nM]	Friedman p-value	Dunn's multiple comparison	
	0, baseline	345.6	(129.2 - 906.2)	0.0003	0 vs 4	*
Tam	4	605.7	(98.3 - 1051.2)		0 vs 8	**
	8	654.0	(114.8 - 1066.8)		4 vs 8	ns
	0, baseline	18.3	(8.9 - 26.9)	0.0004	0 vs 4	***
Tam-NO	4	41.7	(3.7 - 61.0)		0 vs 8	*
	8	31.3	(4.4 - 63.7)		4 vs 8	ns
	0, baseline	628.5	(282.9 - 1913.3)	0.0006	0 vs 4	**
DM-Tam	4	1327.7	(384.0 - 2000.7)		0 vs 8	**
	8	1225.8	(478.1 - 2129.7)		4 vs 8	ns
	0, baseline	94.6	(16.3 - 327.3)	0.0001	0 vs 4	***
DDM-Tam	4	219.7	(62.8 - 424.0)		0 vs 8	**
	8	244.5	(70.4 - 378.6)		4 vs 8	ns
	0, baseline	14.9	(4.5 - 97.9)	0.0388	0 vs 4	ns
Z-Endoxifen	4	24.6	(8.0 - 84.9)		0 vs 8	*
	8	23.5	(7.4 - 102.4)		4 vs 8	ns
	0, baseline	0.60	(0.21 - 3.84)	0.0179	0 vs 4	ns
E-Endoxifen	4	3.31	(0.29 - 35.17)		0 vs 8	*
	8	0.34	(0.23 - 19.75)		4 vs 8	ns
	0, baseline	11.9	(0.4 - 99.2)	0.0121	0 vs 4	*
E + Z-Endoxifen	4	39.0	(8.3 - 89.8)		0 vs 8	*
	8	23.5	(7.4 - 103.2)		4 vs 8	ns
	0, baseline	16.0	(5.7 - 44.6)	0.0023	0 vs 4	ns
Z-4'-OH-DM-Tam	4	24.9	(14.4 - 51.6)		0 vs 8	**
	8	30.8	(19.0 - 55.0)		4 vs 8	ns
	0, baseline	1.48	(0.19 - 7.59)	0.0004	0 vs 4	*
E-4'-OH-DM-Tam	4	3.48	(0.61 - 23.06)		0 vs 8	ns
	8	0.61	(0.35 - 25.41)		4 vs 8	***
	0, baseline	1.36	(0.24 - 9.43)	0.0015	0 vs 4	***
3-OH-DM-Tam	4	3.31	(0.66 - 9.45)		0 vs 8	ns
	8	2.41	(0.81 - 6.70)		4 vs 8	ns
	0, baseline	2.98	(0.83 - 16.07)	0.0007	0 vs 4	***
a-OH-DM-Tam	4	5.89	(1.25 - 17.12)		0 vs 8	*
	8	5.69	(1.21 - 8.00)		4 vs 8	ns
	0, baseline	3.40	(0.81 - 13.24)	0.0096	0 vs 4	ns
4-OH-Tam Z	4	6.25	(1.36 - 12.76)		0 vs 8	*
	8	6.74	(1.64 - 12.85)		4 vs 8	ns
	0, baseline	5.28	(2.30 - 14.91)	0.0047	0 vs 4	*
4'-OH-Tam Z	4	9.36	(3.81 - 18.17)		0 vs 8	*
	8	9.73	(3.70 - 19.38)		4 vs 8	ns
	0, baseline	0.52	(0.13 - 2.68)	0.0028	0 vs 4	**
3-OH-Tam	4	1.09	(0.10 - 2.91)		0 vs 8	ns
	8	0.99	(0.12 - 2.15)		4 vs 8	ns
	0, baseline	1.32	(0.29 - 5.60)	0.0007	0 vs 4	***
a-OH-Tam	4	2.06	(0.26 - 5.22)		0 vs 8	*
	8	1.90	(0.24 - 2.96)		4 vs 8	ns

	0, baseline	3.17	(	0.75	-	12.37)	0.0195	0 vs 4	ns
Z-OH-DDM-Tam	4	4.18	(	0.72	-	11.87)		0 vs 8	*
	8	5.98	(	1.51	-	10.52)		4 vs 8	ns
	0, baseline	0.79	(	0.35	-	2.24)	0.0152	0 vs 4	ns
Tam-N-Gluc	4	1.20	(	0.49	-	4.91)		0 vs 8	*
	8	1.22	(	0.24	-	6.51)		4 vs 8	ns
	0, baseline	0.64	(	0.13	-	4.07)	0.001	0 vs 4	***
E-DM-Tam-4-O-Gluc	4	2.14	(	0.50	-	8.17)		0 vs 8	*
	8	1.88	(	0.46	-	7.52)		4 vs 8	ns
	0, baseline	0.39	(	0.18	-	0.87)	0.0498	0 vs 4	ns
Z-DM-Tam-4-O-Gluc	4	0.52	(	0.16	-	1.90)		0 vs 8	ns
	8	0.42	(	0.16	-	1.54)		4 vs 8	ns
	0, baseline	0.39	(	0.18	-	0.98)	0.2907	0 vs 4	ns
E-Tam-4-O-Gluc	4	0.59	(	0.13	-	1.68)		0 vs 8	ns
	8	0.37	(	0.13	-	1.52)		4 vs 8	ns
MR	0, baseline	0.01965	(	0.0081	-	0.0993)	0.2691	0 vs 4	ns
(Z)-Endoxifen /	4	0.02465	(	0.0060	-	0.0700)		0 vs 8	ns
DM-Tam	8	0.02415	(	0.0051	-	0.0862)		4 vs 8	ns
